# Dissecting the functional and spatial roles of peroxiredoxins as H_2_O_2_ scavengers and redox transducers

**DOI:** 10.1007/s00018-026-06356-1

**Published:** 2026-07-22

**Authors:** Laura de Cubas, Adrian Konopko, Susanna Boronat, José Ayté, Elena Hidalgo

**Affiliations:** 1 Oxidative Stress and Cell Cycle Group, Universitat Pompeu Fabra, C/Dr. Aiguader 88, Barcelona, 08003 Spain; 2 Division of Redox Regulation, German Cancer Research Center (DKFZ), DKFZ-ZMBH Alliance, Heidelberg, 69120 Germany

**Keywords:** Prx, H_2_O_2_ levels, Genetically encoded fluorescent H_2_O_2_ reporter, HyPer7, H_2_O_2_ sensor

## Abstract

Since their discovery in the late 1980s as peroxidases that eliminate hydrogen peroxide (H_2_O_2_), peroxiredoxins (Prxs) have been recognized not only for detoxification but also for roles in redox signaling. In fission yeast, the main Prx, Tpx1, maintains steady-state H_2_O_2_ below toxic levels through its scavenging activity. Under excess peroxides, Tpx1 switches to a signaling role, transmitting oxidative signals to the transcription factor Pap1. Dissecting the relative contributions of Tpx1’s peroxidase and sensor functions is challenging. To address this, we used a synthetic biology strategy to separate these dual roles. Tpx1 or catalase (Ctt1) was expressed in distinct subcellular compartments, and their capacity to remove H_2_O_2_ and activate Pap1 was assessed. Both scavengers can complement the growth defects of cells lacking Tpx1 in high oxygen. Combined with H_2_O_2_ biosensors, our results show that Tpx1 efficiently controls H_2_O_2_ levels from any compartment, whereas Ctt1 does not. However, cells expressing mitochondrial- or nuclear-targeted Tpx1 display reduced oxidative stress tolerance because Pap1 activation requires cytosolic Tpx1. Overall, Tpx1 is a highly effective peroxidase throughout the cell, but its subcellular localization is essential for proper redox signaling regulation.

**Supplementary Information:**

The online version contains supplementary material available at https://doi.org/10.1007/s00018-026-06356-1.

## Introduction

Reactive oxygen species (ROS) are produced in all oxygen-consuming organisms. The main site of production of ROS is the mitochondria [1]. One of these species, H_2_O_2_, can damage biomolecules but also participates in signaling events; it can cross membranes to higher or lower extent and therefore antioxidant activities are localized in different subcellular compartments to contribute to the final peroxide steady-state levels and to H_2_O_2_ signaling fluxes [2–4].

The main families of H_2_O_2_ scavengers are catalases, thioredoxin peroxidases (peroxiredoxins; Prxs) and glutathione peroxidases. While catalases use a heme group to catalyze the dismutation of two molecules of H_2_O_2_ into oxygen and H_2_O [5], the active sites of Prxs and glutathione peroxidases feature a redox-active cysteine (Cys) residue (the peroxidatic Cys), which undergoes oxidation to a sulfenic acid by H_2_O_2_; this group then reacts with another Cys residue, named the resolving Cys to form a disulfide that is subsequently reduced by an appropriate electron donor to complete a catalytic cycle [6, 7]. Eukaryotic organisms express a different collection of H_2_O_2_ scavengers, and members of each of these families are expressed from the same or different genes to give rise to differentially localized isoforms.

We and others have performed a genetic characterization of the repertoire of H_2_O_2_ scavenging activities in the fission yeast *Schizosaccharomyces pombe*. The main Prx in *S. pombe*, Tpx1, has high sensitivity for its substrate, H_2_O_2_, and it is expressed at very high number of molecules per cell [8] as to control the low levels of peroxides produced during aerobic growth: cells lacking this protein are only able to grow on solid plates under low-oxygen conditions [9]. Catalase is the next barrier of detoxification when the steady-state levels of peroxides are increased in Δ*tpx1* cells [10]. Furthermore, cells lacking Ctt1 are very sensitive to extracellularly added peroxides, and over-expression of Ctt1 is sufficient to suppress the sensitivity to oxidative stress of cells lacking the antioxidant transcription factors Pap1 or Atf1 [10]. *S. pombe* also expresses other two Prxs, Pmp20 and Bcp1, of unclear participation in H_2_O_2_ homeostasis [10]. Gpx1, the only glutathione peroxidase encoded by the *S. pombe* genome, only has a minor role, if any, when extracellular peroxides are added [10] or during the stationary phase [11].

While in other eukaryotes different Prx isoforms are expressed at different subcellular compartments, Tpx1 seems to display only cytosolic localization. By using the H_2_O_2_ biosensor HyPer7 expressed at either the cytosol or the matrix we have recently proposed that the steady-state levels of peroxides at the mitochondrial matrix, are significantly higher than those at the cytosol [12]. This steep gradient between the main site of ROS production and the cytosol is caused by Tpx1, which very efficiently consumes most peroxides diffusing out of the mitochondria. Indeed, cells lacking Tpx1 display high levels of peroxides in both compartments [12].

Regarding the role of Tpx1 in cell signaling, this Prx of cytosolic localization has been demonstrated to be essential for activation of the transcription factor Pap1 (pombe AP-1) [13, 14]. As shown before for other transcription factors, Pap1 is blind to peroxides in cells lacking the upstream component of this redox signaling cascade, Tpx1. Two models have been described for H_2_O_2_-dependent signal transduction process: a sulfenic acid-to thiol redox relay (this is the case of the Gpx3-Yap1 in budding yeast [15]) or a disulfide-to-thiol transfer [16]. The genetic and biochemical characterization of the Tpx1-Pap1 redox relay suggests that oxidized Prx, once the cellular reducing system becomes transiently overcome, can transfer the redox-signal to Pap1 through a disulfide-to-thiol relay, causing the formation of two intra-molecular disulfide bonds in Pap1 which promote the nuclear accumulation of the transcription factor and the activation of its antioxidant gene expression program [17, 18]. Tpx1 has also been proposed to positively regulate the activation of the mitogen activated protein (MAP) kinase Sty1, although in this case the effect is only partial (cells lacking Tpx1 can still activate the MAP kinase to a lower extent that in wild-type cells) [19].

As explained above, the lack of Tpx1 causes significant growth defects under aerobic conditions, due to the lack of H_2_O_2_ scavenging capacity; under this situation, it is not easy to decipher the contribution of Tpx1 as a sensor of antioxidant cascades in the cell’s tolerance to oxidants. Here, we have taken a synthetic biology approach to construct strains expressing Tpx1 exclusively in the cytosol or in the matrix of fission yeast cells, and we have analyzed the effects on: (i) basal H_2_O_2_ levels within each compartment; (ii) phenotypic consequences on aerobic growth; (iii) scavenging capacity upon H_2_O_2_ treatments added at the extracellular milieu; and (iv) tolerance to H_2_O_2_ on plates. Our study concludes that cells expressing Tpx1 at either the cytosol or the mitochondria can accomplish wild-type levels of H_2_O_2_ under basal or treated conditions. Nevertheless, physical separation of the two-component redox system, Tpx1-Pap1, fully inactivates the relay and yields cells sensitive to oxidative stress.

## Materials and methods

### Fission yeast growth media and genetic manipulations

For all the experiments, cells were grown in rich media (YE5S) or minimal medium (MM) at 30 °C as previously described [20]. Fluorimeter experiments were performed with filtered MM supplemented with adenine when required at a final concentration of 225 mg/L. The strains used in this study and their genotypes are provided in Table 1. Strains EP159 (*h*^*−*^ *leu1-32 ura4D-18 pap1-GFP::natMX6*) and SG1 (*h*^*+*^ *leu1-32 ade6-M210 ura4-D18 tpx1::natMX6*) were constructed by exchanging the *kanMX6* antibiotic cassettes from MC42 and AV42, respectively, by recombination. Strain SG7 (*h*^*+*^ *ura4-D18 tpx1::kanMX6*) was obtained by mating strain 972 with AV42. LC106 (*h*^*?*^ *ura4-D18 pap1-GFP::natMX6 tpx1::kanMX6)* was created by mating of strains SG7 and EP159. Strain JA3534 (*h*^*+*^ *leu1-32 ade6-M210 ura4-D18 tpx1::natMX6 kanMX6::sty1’-NLS-tpx1::ura4*) was done by transformation of strain SG1 with plasmid pAY1280. To create a strain expressing Pap1.∆NLS-GFP, first a fragment coding for Pap1.∆NLS was amplified and transformed into IC1, yielding LM69 (*h*^*−*^ *ura4-18 leu1-32 pap1. ∆NLS*). LM69 was then transformed with a *GFP-natMX6* cassette (from PCR amplification of from pFA plasmid [27] and primers of the 3’ end of *pap1*) creating LM102.∆NLS (*h- ura4-18 leu1-32 pap1.∆NLS-GFP::natMX6*). Finally, LM102.∆NLS was crossed with strains AV42 or JA3534 originating JA3621 (*h*^*?*^ *leu1-32 ura4-D18 tpx1::kanMX6 pap1.ΔNLS-GFP::natMX6*) and JA3622 (*h*^*?*^ *leu1-32 ura4-D18 tpx1::natMX6 pap1.ΔNLS-GFP::natMX6 kanMX6::sty1’::NLS-tpx1::ura4*), respectively. Strains SG7 and AV42 were transformed with the integrative plasmid p830’ (coding for Tpx1 under the control of the *sty1* promoter) yielding LC97 (*h*^*+*^ *ura4-D18 tpx1::natMX6 sty1’::tpx1::ura4*) and AK1 (*h*^*+*^ *ura4-D18 leu1-32 ade6-M216 tpx1::kanMX6 sty1’::tpx1::ura4*). Finally, strain MFC24 (*h*^*?*^ *ura4-D18 sdh2-mcherry::natMX6*) was transformed with plasmid p900’ to generate MFC30 (*h*^*?*^ *ura4-D18 sdh2-mcherry::natMX6 sty1’::MTS*^*aco1*^*-tpx1-GFP::ura4*).

**Table 1:** Strains used in this study

Strain	Genotype	Origin
972	*h*^*−*^	[21]
975	*h*^*+*^	[21]
SG4	*h*^*+*^ *tpx1::natMX6*	[22]
AV25	*h*^*−*^ *pap1::kanMX6*	[23]
SG7	*h*^*+*^ *tpx1::kanMX6 ura4-D18*	This work
PN513	*h*^*−*^ *ura4-D18 leu1-32*	Paul Nurse lab
AV42	*h*^*+*^ *tpx1::kanMX6 leu1-32 ura4-D18 ade6-M210*	[9]
IC132	*h*^*+*^ *pap1-GFP:: kanMX6*	[24]
LC106	*h*^*?*^ *pap1-GFP::natMX6 tpx1::kanMX6 ura4-D18*	This work
LC97	*h*^*+*^ *tpx1::natMX6 sty1’::tpx1::ura4 ura4-D18*	This work
IC1	*h*^*−*^ *pap1::ura4*^*+*^ *ura4-D18 leu1-32*	[25]
LM69	*h*^*−*^ *pap1.* Δ*NLS ura4-18 leu1-32.*	This work
LM102∆NLS	*h*^*−*^ *pap1∆NLS-GFP::natMX6 ura4-18 leu1-32*	This work
JA3621	*h*^*?*^ *tpx1::kanMX6 pap1.ΔNLS-GFP::natMX6 leu1-32 ura4-D18*	This work
JA3622	*h*^*?*^ *tpx1::natMX6 pap1.ΔNLS-GFP::natMX6 kanMX6::sty1’::NLS-tpx1::ura4 leu1-32 ura4-D18*	This work
SG1	*h+ tpx1::natMX6 leu1-32 ade6-M210 ura4-D18*	This work
JA3534	*h+ tpx1::natMX6 kanMX6::sty1’-NLS-tpx1::ura4 leu1-32 ade6-M210 ura4-D18*	This work
AK1	*h*^*+*^ *ura4-D18 leu1-32 ade6-M216 tpx1::kanMX6 sty1‘::tpx1::ura4 with episomal p728*	This work
MC42	*h*^*−*^ *pap1-GFP::kanMX6 ura4-D18 leu1-32*	[25]
EP159	*h*^*−*^ *leu1-32 ura4D-18 pap1::GFP::natMX6*	This work
MFC35	*h*^*?*^ *ura4-D18 sdh2-mcherry::natMX6 sty1’::MTS*^*aco1*^*-ctt1-GFP::ura4*	[26]
MFC30	*h*^*?*^ *ura4-D18 sdh2-mcherry::natMX6 sty1’::MTS*^*aco1*^*-tpx1-GFP::ura4*	This work

### Plasmids

The plasmids used in this study to express fusion proteins are provided in Table 2. Plasmids pREP.42x [28], p790 [12], p718 [12], and p730 [12] were previously described. p821 was created by Gibson assembly method from PCR fragments encoding *sty1* promoter, and the mitochondrial targeting sequence (MTS) of Aco1, as previously described [12], and both were cloned into p123.42x [13] allowing the expression of episomal MTS-Tpx1. Plasmid p829 was done by digesting p821 to release the MTS and filling ends with Klenow polymerase and then re-ligate, to allow the expression of episomal Tpx1. Insert from p829 was then cloned into pAY1031 yielding p830’ coding for Tpx1 integrative. Cytosolic expression of Ctt1 was obtained by first cloning of *ctt1* from p418.41x [10] in the episomal ura-selection vector pREP.42x [28]. The promoter was then changed to the *sty1* promoter, yielding p464’. Finally, plasmid pAY1280 coding for NLS-Tpx1 was constructed by annealing oligos for NLS and cloning *kanMX6* cassette into an integrative *ura4* plasmid coding for Tpx1. To express MTS-Tpx1-GFP, GFP was PCR-amplified and cloned by Gibson method in the 3’ end of the coding sequences of p832’, yielding plasmid p900’.

**Table 2:** Plasmids used in this study

Plasmid	Description	Origin
pREP.42x	empty vector with *nmt42x prom* (episomal)	[28]
p123.42x	*nmt42 prom::HA-tpx1* (episomal)	[13]
p418.41x	*nmt41x prom::ctt1* (episomal)	[10]
p418.42x	*nmt42x prom::ctt1* (episomal)	[29]
p464	*sty1 prom::ctt1* (episomal)	[29]
p790	*sty1 prom::MTS*^*aco1*^*-ctt1* (episomal)	[12]
p821	*sty1 prom:: MTS*^*aco1*^*-tpx1* (episomal)	This work
p829	*sty1 prom::tpx1* (episomal)	This work
p728	*sty1 prom::HyPer7* (episomal)	[12]
p730	*sty1 prom::MTS*^*aco1*^*-HyPer7* (episomal)	[12]
p830’	*sty1 prom::tpx1* (integrative)	This work
pAV0133	empty vector for integration in *ura4* (integrative)	[30]
pAY1280	*kanMX6-sty1 prom::NLS*^*SV40*^*-tpx1* (integrative)	This work
p832’	*sty1 prom::MTS*^*aco1*^ *-tpx1* (integrative)	[26]
p900’	*sty1 prom::MTS*^*aco1*^*-tpx1-GFP* (integrative)	This work

### H_2_O_2_ survival assays

Assays were performed as previously described [31]. Briefly, strains were grown at 30 °C in YE5S or MM until they reached logarithmic phase, at an OD_600_~0.5. The same number of cells (10^5^ and 1/10 serial dilutions) in 3 µl was spotted on YE5S or MM agar plates, as indicated, with or without the indicated concentration of H_2_O_2_. The spots were allowed to dry and the plates were incubated at 30 °C for 2–4 days under aerobic conditions or inside an anaerobic chamber, with low oxygen tension, as described before.

### Growth of strains expressing HyPer7 or MTS-HyPer7 for fluorescence determination

For wild-type backgrounds, standard MM-based early stationary phase pre-cultures were diluted in filtered MM to reach an OD_600_ of 1 after 4–5 duplications. The fluorescence of 190 µl of these cultures, at an OD_600_ of 1, was directly monitored in 96-well plates in a FLUOstar OMEGA (BMG Labtech) as described below.

### In vivo measurement of basal and induced oxidation of HyPer and roGFP-derivatives

The oxidation of genetically encoded reporters was monitored using a fluorescence plate reader FLUOstar OMEGA (BMG Labtech). We used excitation filters of 400 − 10 and 485BP12, combined with emission filter EM520. The experiments were performed as previously described [12]. The graphs depict the probe *ox*idation *d*egree (OxD) before treatment (OxD_0_) or after treatment over time; 0 corresponds to treatment with 50 mM dithiothreitol (DTT), and 1 corresponds to treatment with 1 mM H_2_O_2_. All experiments were performed in biological triplicates using cells obtained from three independent cell cultures.

### TCA extracts and immunoblot analysis

TCA extracts with alkylating agents followed by non-reducing polyacrylamide electrophoresis was obtained as described before [13]. Briefly, yeast cells were grown in MM until an OD_600_ of 0.5 was reached. Trichloroacetic acid (TCA) was added to a final concentration of 10%. Cells were pelleted and washed in 20% TCA. The pellets were resuspended in 100 µl of 12.5% TCA and lysed by vortexing in the presence of glass beads. Cell lysates were pelleted, washed in acetone, and dried. Pellets were resuspended in alkylating buffer (0.1 M Tris-HCl pH 8.0, 1 mM EDTA, 1% SDS, 75 mM iodoacetamide), loading buffer with (standard SDS-PAGE) or without DTT (non-reducing SDS-PAGE) was added and samples were boiled for 5 min at 100 °C. Samples were detected by immunoblotting with monoclonal anti-GFP antibody (Takara) or polyclonal anti-Tpx1 antibody [9], as indicated.

### Statistics

For *in vivo* fluorescence probe oxidation quantification, OxD, one independent culture of the strain of interest was grown for each replicate. In all figure panels, values of mean of *n* = 3 are shown, with error bars representing standard deviation (S.D.). When indicated statistics were performed with a Student t-test.

### Data and code availability

Original data such as images and blots have been deposited in Figshare (https://doi.org/10.6084/m9.figshare.31444942).

## Results

### Enhancing cellular H_2_O_2_ scavenging capacity in either the cytosol or the mitochondrial matrix suppresses the aerobic growth defects of Δ*tpx1*

**Fig. 1:**
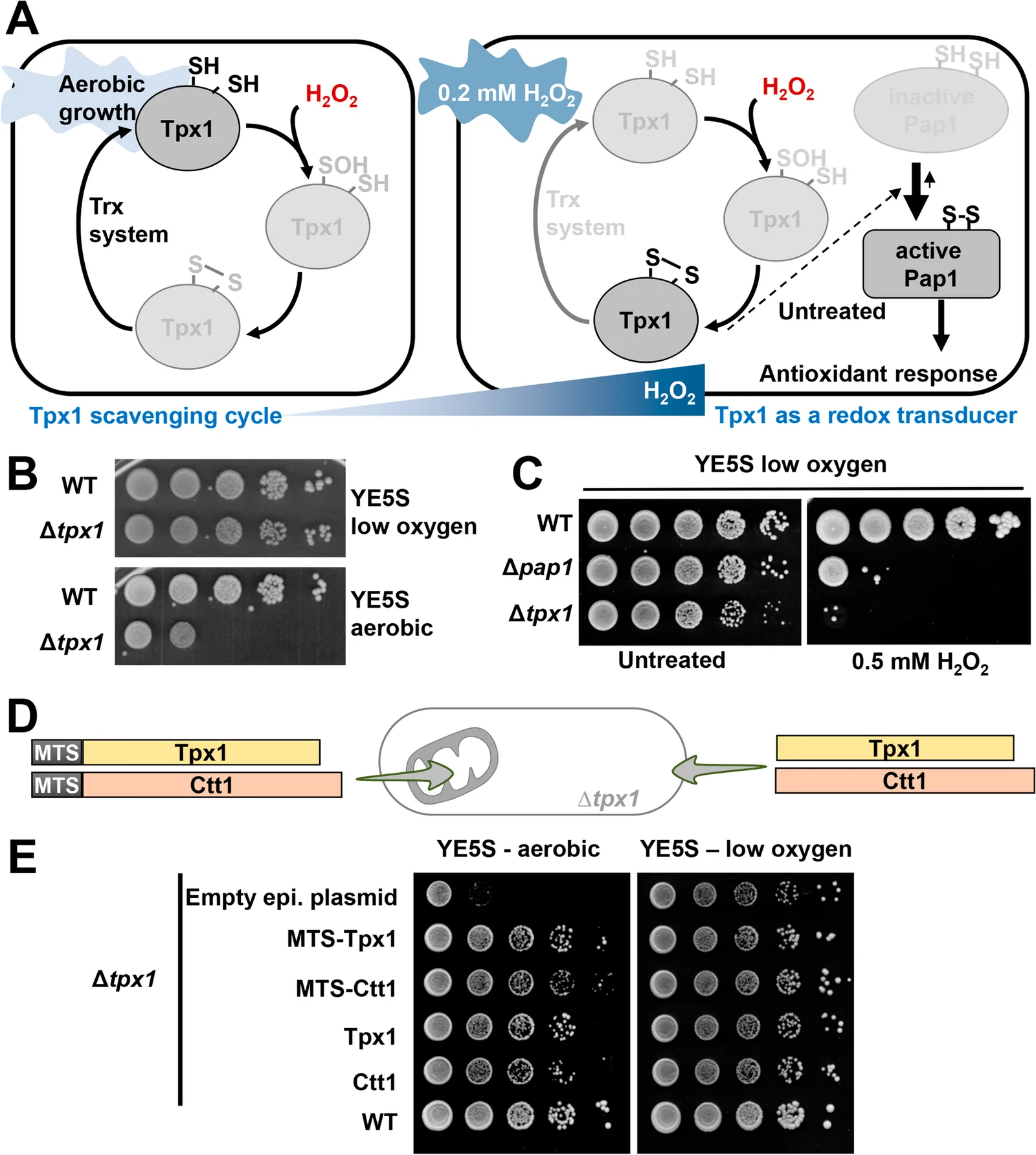
Overexpressing mitochondrial or cytosolic scavengers recover aerobic growth on *∆tpx1* strain. (**A**) Image depicting the dual role of Tpx1. Under aerobic conditions Tpx1 scavenges H_2_O_2_ produced by the cell. When H_2_O_2_ concentration rises an accumulation of oxidized Tpx1 triggers Pap1 oxidation and activation of antioxidant stress response. (**B**) *∆tpx1* can only grow under low oxygen conditions. Strains 972 (WT) and SG4 (*∆tpx1*) were grown in liquid YE5S media, diluted sequentially (number of cells from 10^5^ in 1/10 dilutions), and spotted onto plates to be grown on aerobic or low oxygen conditions. (**C**) Nor *∆pap1* neither *∆tpx1* can survive stress imposition. Strains 972 (WT), SG4 (*∆tpx1*), AV25 (*∆pap1*) were grown and spotted as described in (**B**) onto plates of YE5S supplemented with H_2_O_2_ at the indicated concentrations, and grown under low oxygen conditions. (**D**) Representation of the combination of scavengers used for this study Ctt1 and Tpx1, and their localized expression in cytosol and mitochondrial matrix. (**E**) Complementation of *∆tpx1* with scavengers recovers aerobic growth. Strains 972 (WT), and SG7 (*∆tpx1*) transformed with episomal plasmids pREP.42x (empty plasmid), p821 (MTS-Tpx1), p790 (MTS-Ctt1), p829 (Tpx1) and p464 (Ctt1) were grown in liquid MM media, spotted onto plates and grown under aerobic or low oxygen conditions

As described in the Introduction, Tpx1 scavenges H_2_O_2_ generated during basal aerobic metabolism, likely originating mainly from mitochondria. During aerobic growth, most of the abundant Tpx1 exists as non-covalent dimers, despite being continuously reducing peroxides to maintain physiological H_2_O_2_ levels (Fig. 1A, left panel). When extracellular peroxide concentrations rise, Tpx1 predominantly forms disulfide-linked dimers because oxidation exceeds reduction. Under these conditions, the electron donor capacity may become transiently exhausted, and oxidized Tpx1 shifts toward its signaling role, activating Pap1 (Fig. 1A, right panel). As shown in Fig. 1B, the scavenging function of Tpx1 is readily demonstrated: cells lacking this peroxiredoxin fail to grow aerobically on solid media. To assess Tpx1’s role in Pap1 activation, cells must be plated on media containing added peroxides (Fig. 1C). However, impaired growth in the presence of high H₂O₂ levels under low oxygen conditions may result either from insufficient scavenging capacity or from defective redox signaling to Pap1.

To further investigate the contribution of the main H_2_O_2_ scavengers, Tpx1 and Ctt1, in the cell’s physiology, and to separate the scavenging and signaling roles of Tpx1, we design four strains expressing the scavengers in different sub-cellular locations. Thus, we expressed in strain Δ*tpx1* Ctt1 or Tpx1 from episomal plasmids and under the control of the constitutive *sty1* promoter, either at the cytosol or at the mitochondrial matrix (using the mitochondrial targeting signal (MTS) from Aco1, a matrix resident protein [12]) (Fig. 1D). Of note, the MTS tag is capable of dragging Tpx1 and Ctt1 to the mitochondrial matrix, as previously demonstrated with fluorescence microscopy and subcellular fractionation [26]. The concentrations of the Tpx1 isoforms expressed from these episomal or integrative plasmids were very high (100,000–250,000 copies per cell), based on comparison with endogenous wild-type Tpx1 levels [~ 400,000 copies per cell [8], as shown in Supplementary Fig. 1AB using polyclonal anti-Tpx1 antibodies. Because antibodies against Ctt1 were not available, we used tagged versions of Tpx1 and Ctt1 to compare the levels of these scavengers. This also allowed us to confirm that Ctt1 expression levels were comparable to those of Tpx1, as both proteins were expressed under the control of the same *sty1* promoter (Supplementary Fig. 1 C). Also importantly, we have previously demonstrated that the matrix electron donor network is sufficiently robust to support the scavenging activity of MTS-Tpx1 [32]. As shown in Fig. 1E, the four isoforms of these scavengers could suppress the phenotypic defects on aerobic solid plates of cells lacking Tpx1.

### Ctt1 and Tpx1 have different effects on basal steady-state levels of peroxides

To analyze the molecular bases of the suppression of Δ*tpx1* growth defects by these scavengers, we used the H_2_O_2_ biosensor HyPer7, expressed either at the cytosol or the matrix in these different strain backgrounds. We have shown before that HyPer7 and MTS-HyPer7 expressed in wild-type cells display different basal oxidation levels, OxD_0_, of ∼0 and 0.4 respectively, mainly due to higher relative peroxide levels in the matrix compared to the cytosolic levels [12, 32]. When expressed in strain Δ*tpx1*, HyPer7 and MTS-HyPer7 display OxD_0_ values of ∼0.6 in both compartments, indicating that the peroxide gradient between the matrix and the cytosol is caused by the powerful scavenging at the cytosol exerted by the Prx, and that the H_2_O_2_ toxic levels corresponding to an OxD_0_ of ∼0.6 cause growth defects [12].

**Fig. 2:**
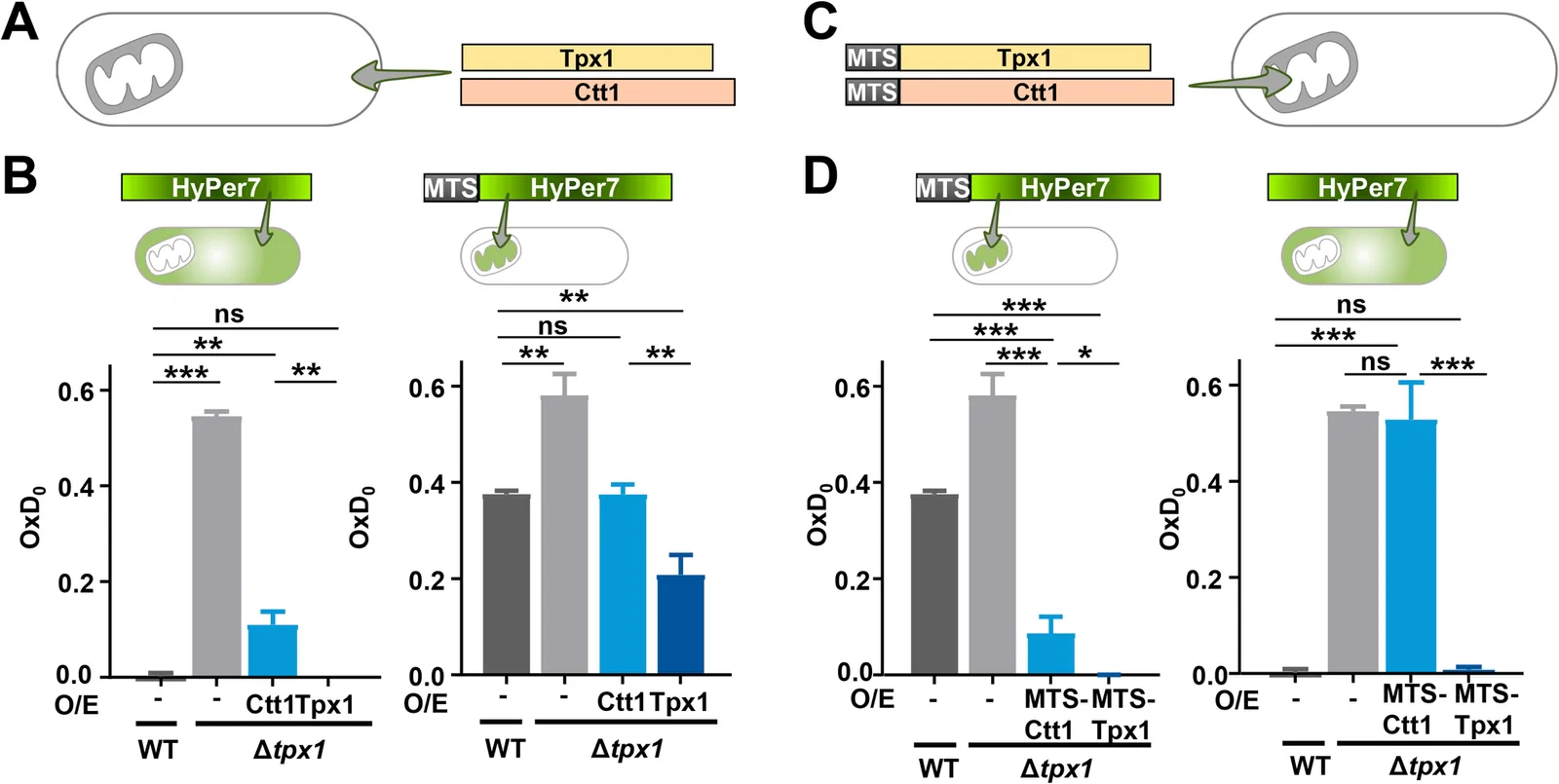
Effect of Ctt1 and Tpx1 isoforms in steady-state H_2_O_2_ levels based on HyPer7 OxD_0_. (**A, B**) Strains PN513 (WT) and AV42 (*∆tpx1*) were transformed with plasmids pREP.42x (empty plasmid), p464 (Ctt1) and p829 (Tpx1) together with p728 (HyPer7) in left panel or with p730 (MTS-HyPer7) in right panel. Bars depicting degree of probe oxidation (amount of probe oxidized per 1) in steady-state conditions (prior to treatment) is shown in the Y-axis (OxD_0_) measuring in MM cultures at an OD_600_ of 1 in 96-well imaging plates, with constant temperature (30 °C) and shaking. For each strain, average data from three biological replicates is shown, with error bars (S.D.). (**C, D**) Strains PN513 (WT) and AV42 (*∆tpx1*) were transformed with plasmids pREP.42x (empty plasmid), p790 (MTS-Ctt1) and p821 (MTS-Tpx1) together with p730 (MTS-HyPer7) in left panel or with p728 (HyPer7) in right panel. Bars representation, experiment and statistics were performed as in (**A**)

We first tested the effect of expressing Tpx1 or Ctt1 in the cytosol of wild-type or Δ*tpx1* cells (Fig. 2A). As shown in Fig. 2B (left panel), the cytosolic OxD_0_ values were almost (with Ctt1) or fully (with Tpx1) restored to wild-type levels, justifying their suppressor capacity. Regarding the matrix, the OxD_0_ values were also decreased by the cytosolic scavengers to levels close to the wild-type background (Fig. 2B, right panel).

When the scavengers were expressed at the matrix (Fig. 2C), the high mitochondrial peroxide levels of cells lacking Tpx1 were significantly (with MTS-Ctt1) or totally (with MTS-Tpx1) diminished, as determined with the OxD_0_ values (Fig. 2D, left panel). On the contrary, MTS-Ctt1 could not restore wild-type levels of H_2_O_2_ in the cytosol, while the OxD_0_ of cytosolic HyPer7 went down to 0 by expression of MTS-Tpx1 (Fig. 2D, right panel). These results demonstrate that augmenting the cellular H_2_O_2_ scavenging capacity with either Ctt1 or Tpx1 can totally alleviate the aerobic growth defects of Δ*tpx1* cells by decreasing peroxides under toxic levels. Nevertheless, each one of the four cell types displayed different levels of H_2_O_2_ at each compartment.

### After adding peroxides to the media, Tpx1 can scavenge intracellular H_2_O_2_ from any compartment, whereas catalase cannot

**Fig. 3:**
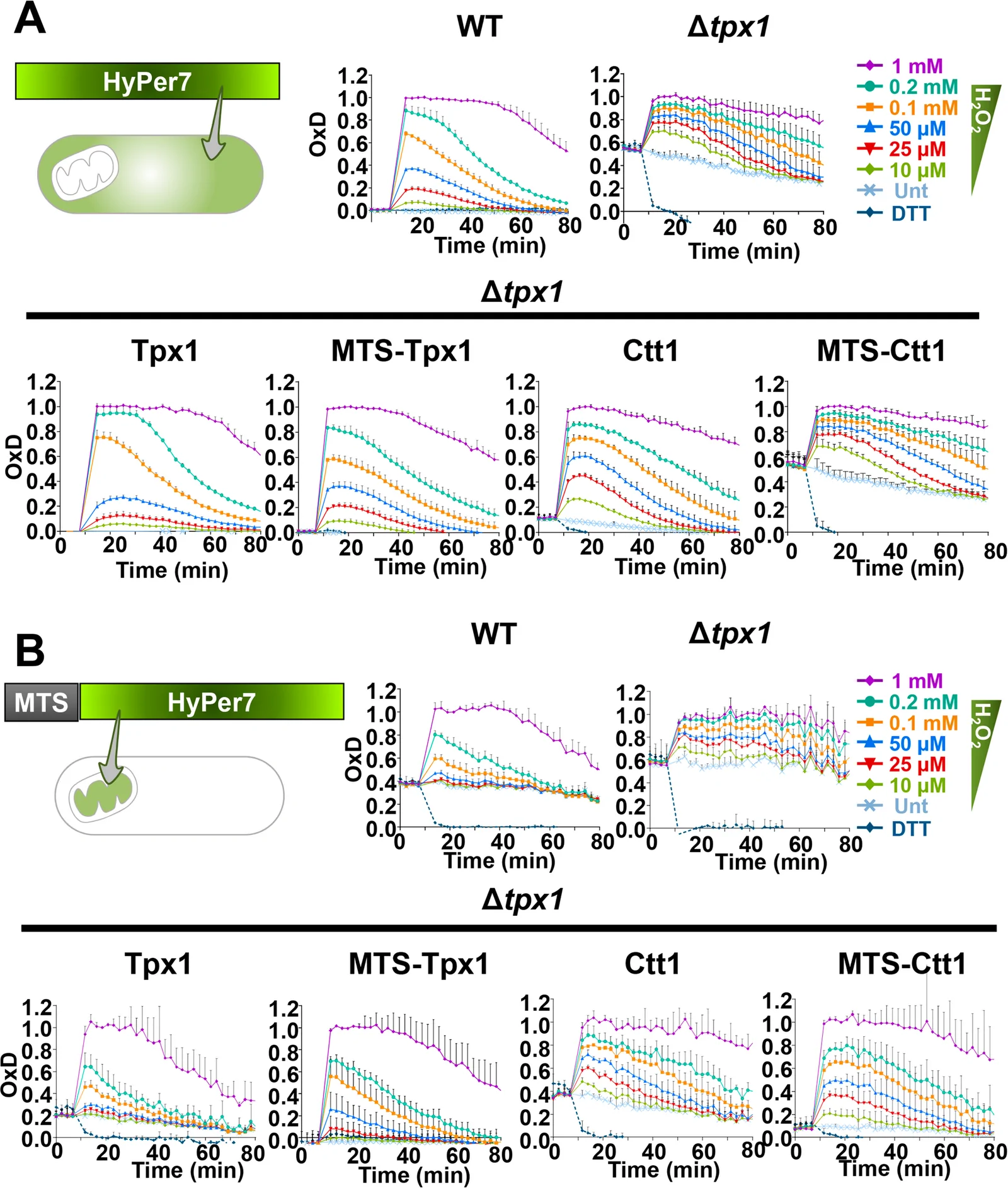
Ctt1 and Tpx1 isoforms display different scavenging capacity upon H_2_O_2_ treatment. (**A**,** B**) Strains PN513 (WT) and AV42 (*∆tpx1*) were transformed with plasmids pREP.42x (empty plasmid), p829 (Tpx1), p821 (MTS-Tpx1), p464 (Ctt1) and p790 (MTS-Ctt1) together with p728 (HyPer7) in (**A**) or p730 (MTS-HyPer7) in (**B**). The indicated extracellular concentrations of H_2_O_2_ were directly added to MM cultures as described in Fig. 2. The degree of probe oxidation (amount of probe oxidized per 1) is indicated in the Y axis (OxD). For each strain, average data from three biological replicates is shown with error bars (S.D.)

We then tested the behavior of each of these four new strains in response to extracellularly added peroxides. Time-dependent probe oxidation following peroxide addition to the growth medium allowed us to assess the extent of initial scavenging in each strain background. The slope of reduction of the biosensor with time depends both on the rates of reduction of the probe by the thioredoxin system [12] and on the rates of intracellular H_2_O_2_ consumption. To estimate the scavenging capacity of the different isoforms, we analyzed the HyPer7 reduction slopes over time: as shown in Fig. 3A (upper panels), HyPer7 recycling was very slow in Δ*tpx1* compared to wild-type cells at all the concentrations of H_2_O_2_ assayed.

The slopes of HyPer7 recycling in Δ*tpx1* cells (from 20 to 80 min in the graphs of Fig. 3) were restored to wild-type levels by expression of Tpx1 or MTS-Tpx1, whereas Ctt1 or MTS-Ctt1 could not modify the kinetics of intracellular peroxides after H_2_O_2_ addition seen in Δ*tpx1* cells: the probe became oxidized, but the reduction slopes at all concentrations assayed were significantly slower than in wild-type cells (Fig. 3A, lower panels).

Regarding matrix levels of peroxides as measured with MTS-HyPer7, both isoforms of catalase (Ctt1 and MTS-Ctt1) had severe problems to improve the reduction slopes of Δ*tpx1* cells. On the contrary, Tpx1 from the cytosol or expressed in the matrix could very much suppress the HyPer7 recycling problems of strain Δ*tpx1* (Fig. 3B, lower panels). In conclusion, unlike catalase, Tpx1 is able to scavenge extracellularly added peroxides from both mitochondria and cytosol.

### Cells expressing MTS-Tpx1 as the sole H_2_O_2_ scavenger are sensitive to excess peroxides because they cannot trigger Pap1 activation

**Fig. 4:**
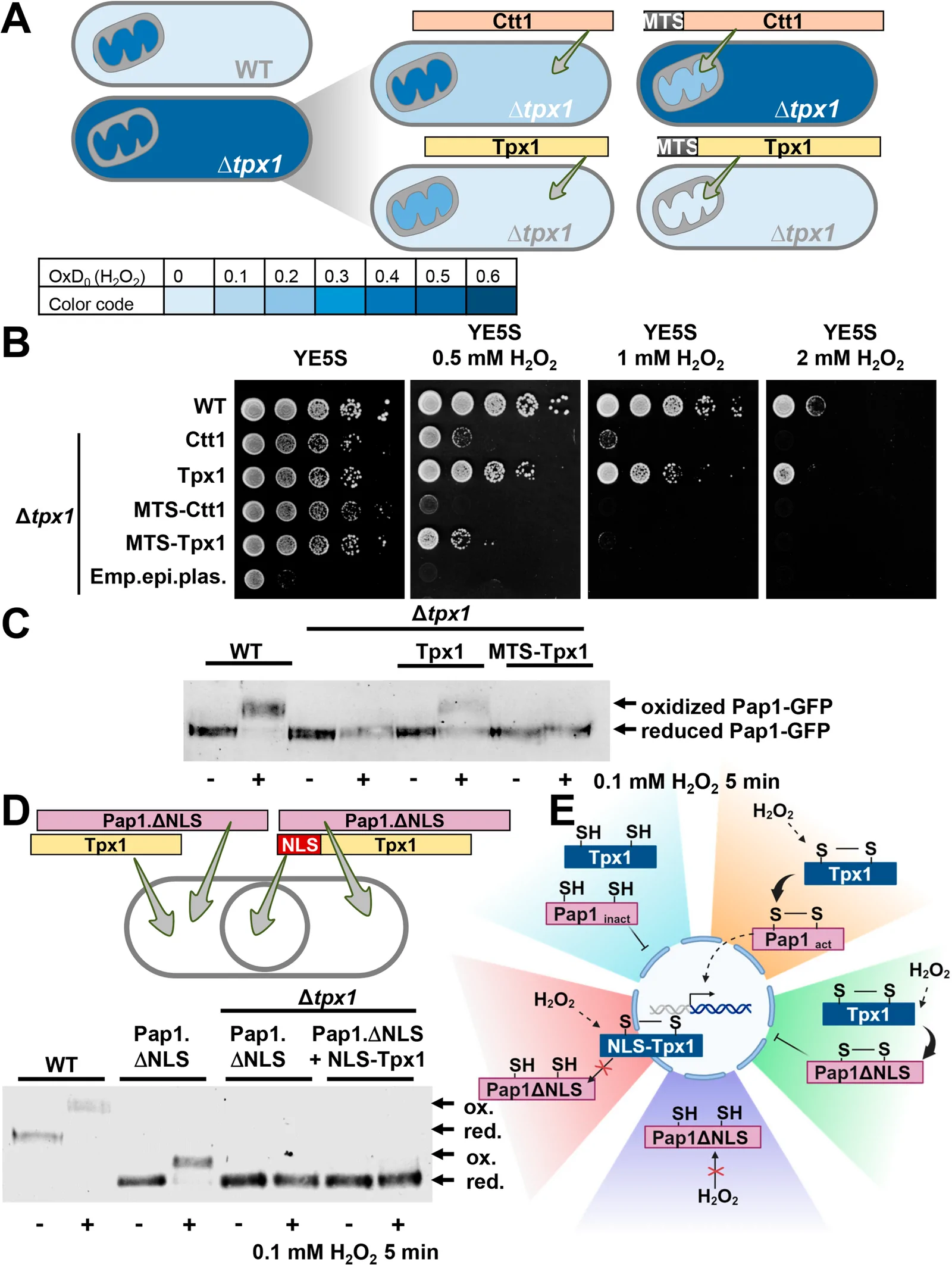
MTS-Tpx1 is sufficient to recover scavenging capacity of *∆tpx1* but not signaling response. (**A**) Scheme representing the H_2_O_2_ levels in cytosol and mitochondrial matrix by complementing ∆*tpx1* with cytosolic or mitochondrial scavengers, based on data of Fig. 2. (**B**) Only overexpression of Tpx1 in *∆tpx1* can suppress its sensitivity to extracellular H_2_O_2_. Strains 972 (WT), and SG7 (*∆tpx1*) transformed with plasmids p419 (Ctt1), p829 (Tpx1), p790 (MTS-Ctt1), p821 (MTS-Tpx1) and pREP.42x (empty plasmid) were grown in liquid MM media and spotted as described in Fig. 1B onto plates supplemented with H_2_O_2_ at the indicated concentrations, and grown under aerobic conditions. (**C**) Oxidation of Pap1 in different strain backgrounds. Strains IC132 (WT, Pap1-GFP), and LC106 (*∆tpx1*, Pap1-GFP) transformed with plasmids pREP.42x (empty plasmid), p829 (Tpx1) or p821 (MTS-Tpx1), were grown on MM treated when indicated with 0.1 mM of H_2_O_2_ for 5 min. TCA extracts were loaded in non-reducing SDS-PAGE gels and western blots were used to determine the redox state of Pap1-GFP (indicated by arrows). (**D**) Scheme of experiment in (**E**), showing localization of the transcription factor Pap1.∆NLS in combination with cytosolic or nuclear Tpx1. Co-localization of Tpx1 and Pap1 is needed for signal transduction. Strains IC132 (WT *pap1-GFP*), LM102. ∆NLS (*pap1.∆NLS-GFP*), JA3621 (*∆tpx1 pap1.∆NLS-GFP*) and JA3622 (*∆tpx1 pap1.∆NLS-GFP* + *NLS-tpx1*) were grown in YE5S and experiments performed as explained in (**C**). (**E**) Figure depicting the different approaches to get or not Pap1 oxidation and how that affect stress response activation

We have shown that the four genetic backgrounds displayed different steady-state levels of peroxides in the cytosol and the matrix (low levels of H_2_O_2_ are represented in light blue and high levels in dark blue in Fig. 4A, based on the OxD_0_ of HyPer7 and MTS-HyPer7 as experimentally determined in Fig. 2B). Furthermore, analysis of H_2_O_2_ scavenging capacity of these four synthetic strains (expressing Ctt1 or Tpx1 in only the cytosol or the matrix) suggested that both Tpx1 isoforms, expressed at the cytosol OR the matrix, could provide sufficient scavenging activity as to render wild-type tolerance to extracellular peroxides. On the contrary, the strains expressing the catalase isoforms might not provide sufficient defense against extracellular peroxides, based on their biosensor oxidation graphs resembling those of the Δ*tpx1* background (Fig. 3).

We tested the phenotypic consequences of localized expression of scavengers by spotting our four strains (together with the wild-type and Δ*tpx1* strains as controls) on aerobic plates containing or not increasing concentrations of H_2_O_2_. Cells lacking Tpx1 were as sensitive to oxidative stress when carrying an empty plasmid or when transformed with expression plasmids for Ctt1 or MTS-Ctt1 (Fig. 4B). As expected, over-expression of cytosolic Tpx1 yielded cells with wild-type tolerance to peroxides: this genetically-engineered strain is very similar to an original wild-type background, although with slightly lower levels of Tpx1 (even though the transgene is located in an episomal plasmid with ~ 7–8 copies per cell [33], it is expressed from the *sty1* promoter instead of from the endogenous *tpx1* promoter, which is ~ 30 times stronger based on proteomic data [8]). However, even though MTS-Tpx1-expressing Δ*tpx1* cells were capable of detoxifying extracellular peroxides to similar extent as a wild-type background (see Fig. 3), they were almost as sensitive to peroxides as cells carrying the empty plasmid (Fig. 4B).

We then monitored the ability of MTS-Tpx1 to transduce the redox signal to Pap1 upon peroxide addition. As shown by western blot, only cells expressing Tpx1 in the cytosol were capable of triggering Pap1 disulfide formation and activation (Fig. 4C). Our results indicate that the role of Tpx1 as a redox transducer to Pap1— which cannot occur if the two proteins are physically separated— is essential for tolerance to oxidative stress.

In the same line, we constitutively expressed Tpx1 in the nucleus, by tagging it with a nuclear localization signal. The NLS-Tpx1 chimera suppressed the aerobic growth defects of strain Δ*tpx1* (data not shown), but could not trigger activation of a Pap1 isoform lacking its NLS signal and with constitutive cytosolic localization [34] (Fig. 4D, lower panel). We conclude that while Tpx1 is equally able to scavenge low levels of peroxides from any subcellular localization, the presence of Pap1 in the cytosol explains why *S. pombe* cells express their main active Prx in this compartment (Fig. 4E).

### High levels of Tpx1 are required for tolerance to extracellular peroxides

**Fig. 5:**
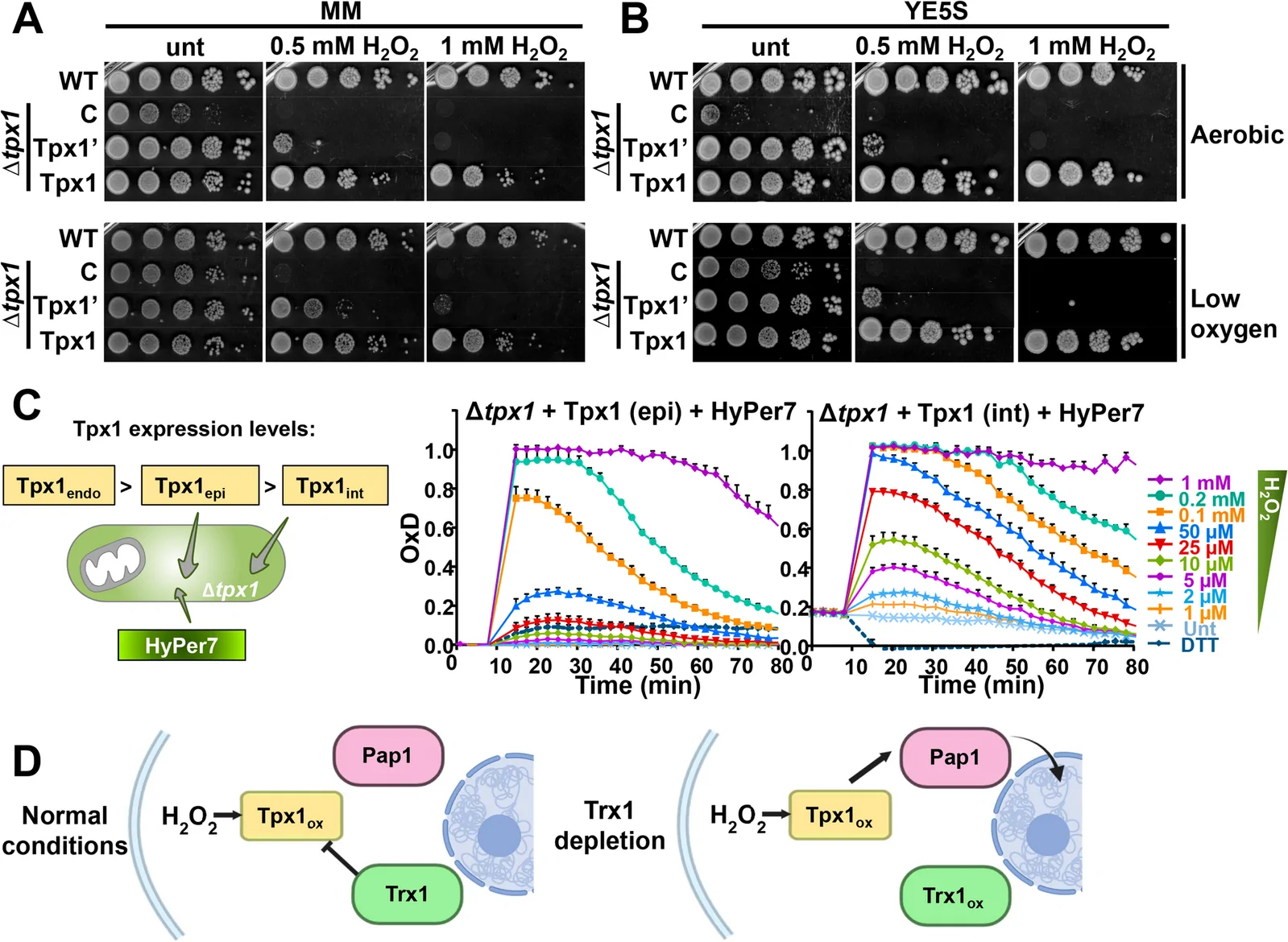
High levels of Tpx1 are required to provide sufficient scavenging capacity. (**A**) Tpx1 dependent survival upon stress imposition. Strains 972 (WT), SG7 (*∆tpx1*) complemented with empty plasmid pREP.42x (*∆tpx1*, C) or p829 (*∆tpx1*, Tpx1 episomal), and LC97 (*∆tpx1*, Tpx1’ integrative). Cells were grown in MM and experiment carried out as described in Fig. 1B. Cells were spotted in MM or YE5S plates containing the indicated concentrations of H_2_O_2_ (**B**) Same as in (A), but cells were grown in YE5S. (**C**) Scheme proposing the analysis of the effect different expression levels of Tpx1 on H_2_O_2_ scavenging based on HyPer7 oxidation. Strains HM123 (WT), AV42 transformed with episomal plasmid p829 [*∆tpx1 +* Tpx1 (epi)] and AK1 [*∆tpx1 +* Tpx1 (int)] were transformed with p728 coding for HyPer7. Experiment was performed as described in Fig. 2. Average of three biological replicates is shown with error bars (S.D.). (**D**) Scheme depicting Pap1 activation by oxidized Tpx1 only when Trx1 function as an electron donor is transiently overwhelmed

Given that Tpx1 is a highly abundant protein in *S. pombe* (more than 400,000 copies per cell) [8], we tested whether reduced protein levels were sufficient to sustain aerobic growth and confer tolerance to oxidative stress. We expressed Tpx1 to different levels in the cytosol of Δ*tpx1* cells, by using integrative or episomal plasmids (in fission yeast, episomal plasmids are replicated at ~ 7–8 copies per cell [33]). As shown in Fig. 5A (MM plates) and Fig. 5B (YE5S plates), the aerobic growth defects of cells lacking Tpx1 were suppressed by either plasmid, but only cells transformed with the episomal plasmid displayed wild-type tolerance to peroxides.

We then compared the H_2_O_2_ detoxifying activity of Tpx1 expressed from an episomal plasmid (left panel of Fig. 5C) or from an integrative plasmid (right panel of Fig. 5C), using again the HyPer7 biosensor. We determined that Δ*tpx1* cells expressing cytosolic Tpx1 from the integrative plasmid could largely reduce the steady-state levels of H_2_O_2_, decreasing the HyPer7 OxD_0_ from 60% of a of the Δ*tpx1* background to 20%. However, the low levels of Tpx1 arising from this integrative plasmid provided very limited peroxide scavenging activity upon H_2_O_2_ addition, and the slopes of HyPer7 reduction/recycling resembled those of cells lacking Tpx1 (see Fig. 3). This result suggest that Tpx1 is a sensitive but not very efficient enzyme regarding peroxide detoxification, and that explains why this peroxiredoxin is one of the proteins with higher copy number per cell in fission yeast [8].

## Discussion

Eukaryotic cells express a complex battery of peroxide scavengers, which vary in their composition, the concentration of the different enzymes and the sub-cellular localization. We have used here fission yeast, with a limited collection of H_2_O_2_ scavengers, to test the consequences of expressing catalases or Prxs in the cytosol or in the main site of ROS production, the mitochondrial matrix. Two of these engineered strains, expressing Ctt1 in either the cytosol or the matrix, display abnormal responses to extracellular peroxides, demonstrating the importance of the powerful Prx-dependent scavenging in wild-type tolerance to oxidative stress. The other two strains, expressing Tpx1 in the cytosol or the matrix, have excellent responses to extracellularly added H_2_O_2_, suggesting that this Prx is a potent H_2_O_2_ scavenger which can control peroxide levels from compartments different from its natural localization. However, its physical separation from downstream components of a signaling cascade can jeopardize wild-type tolerance to peroxides: MTS-Tpx1 cannot activate the transcription factor Pap1 (Fig. 5D) and therefore cells are sensitive to H_2_O_2_.

While all four engineered strains, expressing scavengers in a Tpx1 depleted background, could suppress the aerobic growth defects of strain Δ*tpx1*, their responses to extracellular peroxides were dramatically different. Ctt1 and MTS-Ctt1 could slightly improve H_2_O_2_ scavenging of cells lacking Tpx1 in the respective location where they were expressed, and this effect was limited to such compartment. This result suggest that Ctt1 can scavenge peroxide concentrations over a certain threshold, and it is blind to concentrations below; thus, low H_2_O_2_ levels that are not detected by Ctt1 can diffuse and reach other sub-cellular locations. In some sense, Ctt1 would work as a floodgate, which only starts working when the concentration of its substrate is sufficiently high. While it remains unclear why cytosolic Ctt1 influences mitochondrial steady-state H_2_O_2_ levels whereas mitochondrial Ctt1 does not similarly affect the cytosolic H_2_O_2_, we speculate that MTS-Ctt1, despite being expressed at a concentration comparable to that of MTS-Tpx1, reduces matrix H_2_O_2_ generated by the ETC but fails to prevent H_2_O_2_ leakage from the intermembrane space into the cytosol. Consequently, overexpression of cytosolic Ctt1 in Δ*tpx1* cells, H_2_O_2_ accumulates in the cytosol because this compartment lacks an efficient scavenging system. In contrast, overexpression of cytosolic Ctt1 in Δ*tpx1* cells, which exhibit elevated H_2_O_2_ levels in both compartments, allows cytosolic Ctt1 to function as a sink, thereby reducing the overall intracellular H_2_O_2_ concentration.

The scavenging capacity of Prxs is radically different: Tpx1 from any location can control the concentration of H_2_O_2_ in other sub-cellular compartments. Prxs consume H_2_O_2_ at the site of production with great efficiency. Therefore, in order to allow peroxide fluxes and redox communication inter-compartments, our experiments suggest that active Prxs should not be located at the source of H_2_O_2_ production; otherwise, peroxide fluxes would be fully inhibited. In our synthetic biology study, we have designed a strain expressing MTS-Tpx1 at the matrix, close to the main site of H_2_O_2_ production; this strain can control matrix and cytosolic H_2_O_2_ levels even better than wild-type cells. Contrary to the expectations, its physical separation from downstream components of signaling cascades, such as Pap1, renders its function useless as a signal transducer, and this strain is very sensitive to extracellular peroxides. Similarly, expression of nuclear-localized Tpx1 in Δ*tpx1* is unable to activate Pap1 upon peroxide addition.

Another important issue regarding Tpx1 expression concerns the protein levels required for efficient peroxide scavenging and redox transduction. Tpx1 is a highly abundant protein, expressed at approximately 440,000 copies per cell [8], and expression of Tpx1 from an episomal plasmid yields sufficient protein levels as to provide wild-type tolerance to peroxides. However, reducing Tpx1 levels by approximately tenfold, achieved through expression from an integrative plasmid, renders cells capable of growing on high oxygen conditions, but sensitive to H_2_O_2_. In conclusion, the only configuration that yields wild-type tolerance to peroxides in *S. pombe* is the expression of a highly abundant peroxiredoxin, Tpx1, in the cytosol of fission yeast cells, where it can effectively perform both scavenging and signaling functions.

## Supplementary Information

Below is the link to the electronic supplementary material.

Supplementary File 1 (PDF 179 KB)

## Data Availability

The data that support the findings of this study are available upon reasonable request to the corresponding author.
