# Supplementary material for: Dissecting the functional and spatial roles of peroxiredoxins as H2O2 scavengers and redox transducers
Source: Cell Mol Life Sci. 2026 Jul 22;83(1):384. doi: 10.1007/s00018-026-06356-1 (PMC13680352; doi:10.1007/s00018-026-06356-1)
Supplement: Supplementary file 1 [file 18_2026_6356_MOESM1_ESM.pdf]

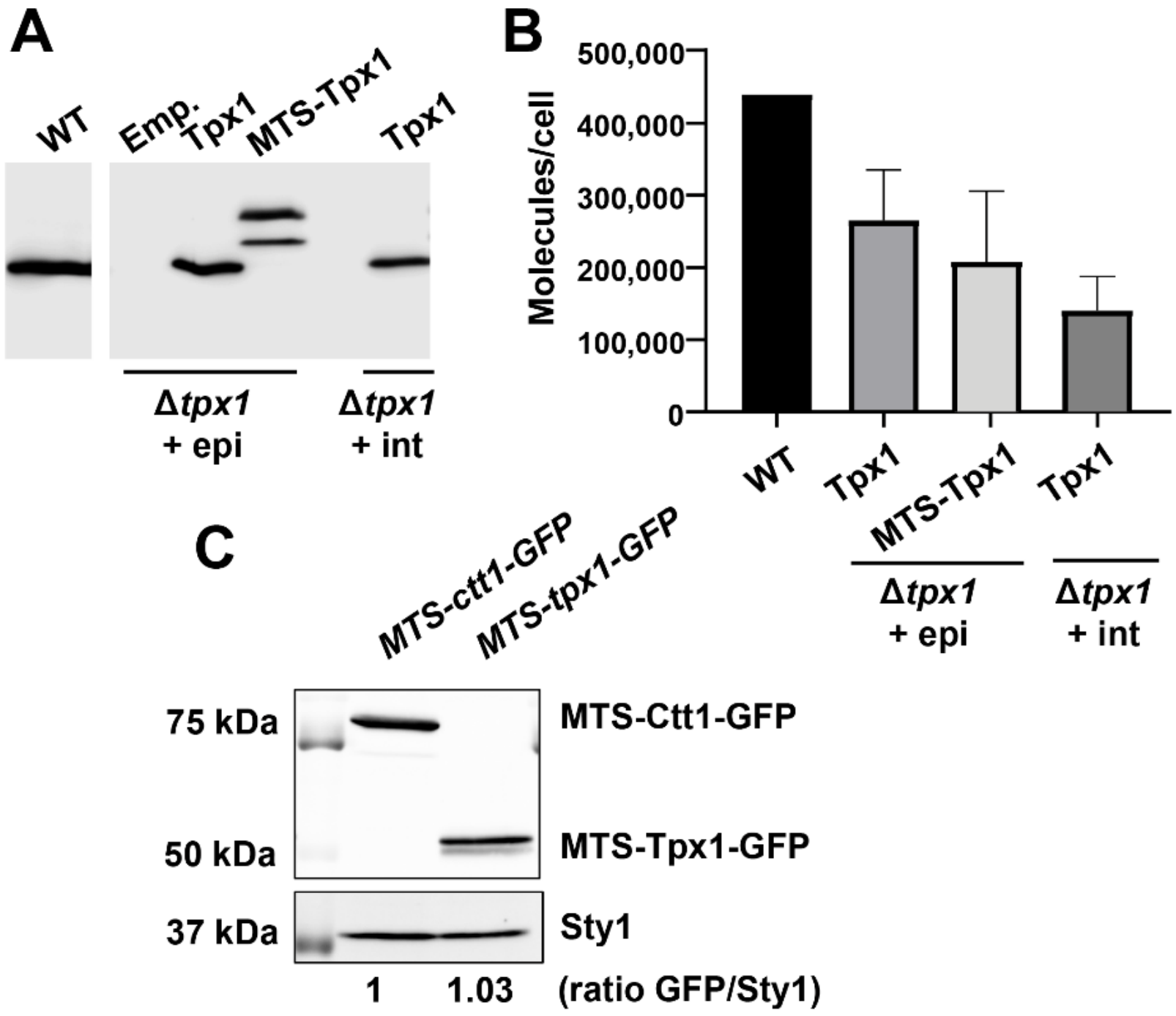

Supplementary Fig. 1. Levels of Tpx1 and Ctt1 expressed from episomal or integrative plasmids from the constitutive *sty1* promoter. (**A**) Western blot showing ectopic Tpx1 expression levels. Strains 972 (WT) and SG7 (Δ*tpx1*), transformed with plasmids pREP.42x (Emp.), p829 (Tpx1 epi), p821 (MTS-Tpx1 epi), and strain LC97 (Δ*tpx1* Tpx1 int) were grown in MM and TCA extracts were run in SDS-PAGE under reducing conditions. (**B**) Quantification of Tpx1 levels. Protein levels were quantified from independent experiments as in A, and levels normalized to WT. (**C**) Western blot of strains MFC35 (expressing MTS-Ctt1-GFP) and MFC30 (expressing MTS-Tpx1-GFP) following same protocol as in A.
